# Molecular and neurological characterizations of three Saudi families with lipoid proteinosis

**DOI:** 10.1186/1471-2350-12-31

**Published:** 2011-02-24

**Authors:** Mustafa A Salih, Khaled K Abu-Amero, Saleh Alrasheed, Ibrahim A Alorainy, Lu Liu, John A McGrath, Lionel Van Maldergem, Yasser H Al-Fakey, Adel H AlSuhaibani, Darren T Oystreck, Thomas M Bosley

**Affiliations:** 1 Departments of Pediatrics (Neurology), College of Medicine, King Saud University, Riyadh, Saudi Arabia; 2 Department of Ophthalmology, College of Medicine, King Saud University, Riyadh, Saudi Arabia; 3 Department of Dermatology, College of Medicine, King Saud University, Riyadh, Saudi Arabia; 4 Radiology Department, College of Medicine, King Saud University, Riyadh, Saudi Arabia; 5 Guy's and St Thomas' NHS Foundation Trust, London, UK; 6 St John's Institute of Dermatology, King's College London (Guy's campus), London, UK; 7 Department of Human Genetics, Université de Liège, Liège, Belgium; 8 Neurology Division, Cooper University Hospital, Camden, NJ

## Abstract

**Background:**

Lipoid proteinosis is a rare autosomal recessive disease characterized by cutaneous and mucosal lesions and hoarseness appearing in early childhood. It is caused by homozygous or compound heterozygous mutations in the *ECM1 *gene. The disease is largely uncharacterized in Arab population and the mutation(s) spectrum in the Arab population is largely unknown. We report the neurologic and neuroradiologic characteristics and *ECM1 *gene mutations of seven individuals with lipoid proteinosis (LP) from three unrelated consanguineous families.

**Methods:**

Clinical, neurologic, and neuro-ophthalmologic examinations; skin histopathology; brain CT and MRI; and sequencing of the full*ECM1 *gene.

**Results:**

All seven affected individuals had skin scarring and hoarseness from early childhood. The two children in Family 1 had worse skin involvement and worse hoarseness than affected children of Families 2 and 3. Both children in Family 1 were modestly mentally retarded, and one had typical calcifications of the amygdalae on CT scan. Affected individuals in Families 2 and 3 had no grossneurologic, neurodevelopmental, or neuroimaging abnormalities. Skin histopathology was compatible with LP in all three families. Sequencing the full coding region of *ECM1 *gene revealed two novel mutationsin Family 1 (c.1300-1301delAA) and Family 2 (p.Cys269Tyr) and in Family 3 a previously described 1163 bp deletion starting 34 bp into intron 8.

**Conclusions:**

These individuals illustrate the neurologic spectrum of LP, including variable mental retardation, personality changes, and mesial temporal calcificationand imply that significant neurologic involvement may be somewhat less common than previously thought. The cause of neurologic abnormalities was not clear from either neuroimaging or from what is known about *ECM1 *function. The severity of dermatologic abnormalities and hoarseness generally correlated with neurologic abnormalities, with Family 1 being somewhat more affected in all spheres than the other two families. Nevertheless, phenotype-genotype correlation was not obvious, possibly because of difficulty quantifying the neurologic phenotype and because of genetic complexity.

## Background

Lipoid proteinosis (LP; MIM 247100) is a rare autosomal recessive disease characterized by cutaneous and mucosal lesions and hoarseness appearing in early childhood[1] that is caused by homozygous or compound heterozygous mutations in the *ECM1 *gene located on chromosome 1q21[2]. This gene encodes a protein that is an important structural component of basement membrane and extracellular matrix[3,4], and loss of protein-protein interactions due to *ECM1 *gene mutations is the likely cause of dermatologic abnormalities including warty skin, scarring, and mucosal thickening[5]. These changes also affect the nasopharynx, tongue, and vocal cords, resulting in severe fibrosis and the hoarseness characteristic of the disorder.

Approximately one third of affected individuals are reported to have mild mental retardation[6], andneuropsychological problems may be more common[7-9]. Other reported neurologic abnormalities include complex partial seizures, memory loss, and emotional difficulties, often beginning in teenage years and progressing from that time onward[6,8]. Calcification of the mesial temporal lobes may be observedon brain imaging and is progressive with age. Pathologic material is limited, and the mechanism of brain injury remains uncertain.

We had the opportunity to study seven individuals from three unrelated consanguineous families who had the typical spectrum of skin, voice, and neurologiccharacteristics of LP with mutations in the *ECM1 *gene.

## Methods

Seven participants from three unrelated Saudi Arabian (Table 1) wereenrolled and all signed informed consent. This study was approved by the institute IRB and adhered to the tenets of the Declaration of Helsinki. Blood samples were collected from all participants and DNA was extracted and stored at -20°C until needed for genetic testing.

**Table 1:** Demographics of three families

Sub ID	Family	Sex	Age at last exam
II-1	1	M	24 years
II-6	1	F	14 years
II-3	2	F	19 years
II-6	2	F	12 years
II-1	3	F	18 years
II-2	3	M	12 years
II-3	3	M	5 years

Intelligence testing was performed by routine assessment on neurologic examination and by using the Stanford Binet Test (Patient II-1, Family 1) and the Wechsler Bellevue Intelligence scale (Patient II-6, Family 1). Patients from each family had skin punch biopsies including epidermis, dermis, and subcutaneous tissue that were fixed in formalin and stained with PAS and PAS/diastase. All affected individualsexcept Patient II-6 of Family 2had brain computed tomography (CT) and either 1.5 or 3.0 Tesla magnetic resonance imaging(MRI). MRI for patients from Family 3 included Susceptibility-Weighted sequences.

The *ECM1 *gene was directly sequenced as described previously in affected individuals, in available first degree relatives, and in 100 chromosomes from healthy individuals of matching ethnicity[2,3]. Nucleotide numbering for mutations reported in all three families was referenced to the NCBI sequence (NG_012062).

## Results

**Figure 1:**
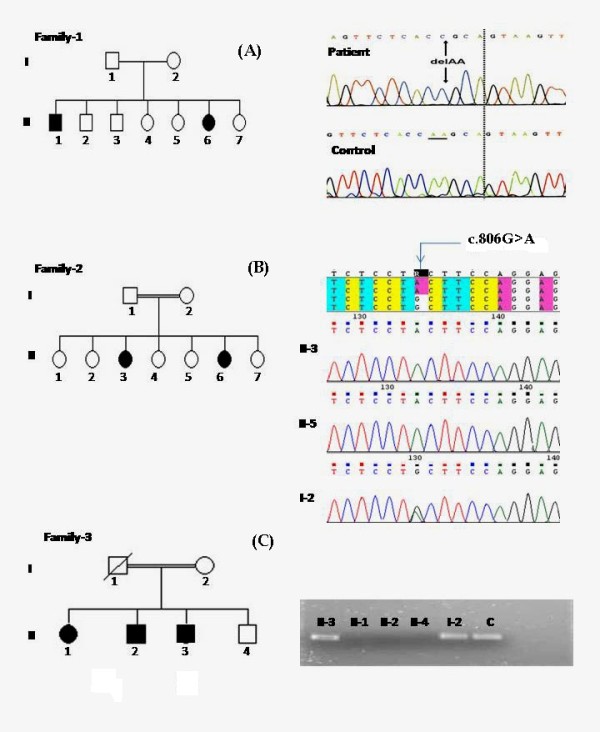
**Pedigrees and Genetic Results**. (a)Family 1 pedigree and *ECM1 *chromatogram showing a homozygous two base deletion detected in affected family members. b) Family 2 pedigree and *ECM1 *chromatogram showing a homozygous base substitution in both affected individuals (II-3 and II-6) and heterozygous base substitution in their mother. Similar heterozygous results were also found in father (II-1) and an unaffected sibling (II-4) but are not shown. c) Family 3 pedigree with photograph of a 1.5% agarose gel showing failed amplification of exons 9 and 10 in all affected individuals but not in their mother or unaffected brother. Forward primer was designed in intron 8 and the reverse primer was designed in intron 10.

Direct sequencing of the *ECM1 *gene inthe two patients (II-1 and II-6) from Family 1 (Figure 1a) identified a novel homozygous two base deletion near the end of exon 8 (c.1300-1301delAA). The deletion occurred at codon 434 and was expected to create a premature stop codon two codons later, likely to result in low levels of all*ECM1 *transcriptsand in severely truncated proteins[3].

Family 2 (Figure 1b) had a novel one-base substitution mutation c.806G > Ain exon 7, which resulted in the replacement of cystine with tyrosine at codon 269 (p.Cys269Tyr). This mutation was detected in homozygous status in both patients (II-3 and II-6) and in heterozygous status in both parents (I-1 and I-2) and one unaffected siblings (II-4). An assessment of the pathologic effect of this mutation utilizing PolyPhen, a tool predicting the possible impact of an amino acid substitution on the structure and function of a human protein using physical and comparative considerations http://genetics.bwh.harvard.edu/pph/[10], predicted this mutation to be probably damaging (i.e., very likely to affect protein structure and/or function). Exon 7 is represented in transcripts *ECM1a *and *ECM1c*[11]but not in the *ECM1b *transcript, and therefore, presumably the *ECM1b *transcript would be normal in this family.

Family 3 (Figure 1c) had a 1163 bp deletion starting 34 bp into intron 8 and encompassing all of exon-9, intron-9, and exon 10, including the termination codon and part of the 3'-UTR. This mutation was detected in homozygous status in all affected individuals in this family (II-1, II-2, II-3), but was not present in an unaffected sibling (II-4). The mother was a carrier of the mutation; the father was deceased but was also expected to be a carrier. This mutation was previously reported in an affected Saudi family from the same tribe thatmay be related by founder effect to the current family[2]. It results in complete loss of exons 9 and 10 from transcripts *ECM1a/c *and *ECM1b *and is predicted to have a deleterious effect on protein structure and function[2].

The two siblings in Family 1 were the only affected individuals in this consanguineous family with seven children (Figure 1A). Patient II-1 was born at eight months gestation and stayed in a neonatal intensive care unit for six weeks, while Patient II-6 was the product of a normal pregnancy and delivery. They each had mild motor delay, walking at age 18 months and talking at approximately age three years. They were modestly mentally retarded with intelligence quotients of approximately 60 and a requirement for special education. Neither had developed seizures or obvious emotional problems when last examined at ages 25 (Patient II-1) and 14 (Patient II-6)years.

Both children had normal skin appearance at birth, but each had an abnormal cry and developed hoarseness by age six months. Patient II-1 had the most severe presentation during early childhood in this group with irregular, indurated, partially coalescent yellowish papules with hypo- and hyperpigmented macular plaques in the lower back and gluteal regions. He developed skin-colored, verrucous papules and nodules on the elbows, knees, and sites of friction and movement including the hands. His tongue was enlarged and woody, hard on palpation, with inability to protrude tongue. The frenulum and oral mucosa were infiltrated, but dentition was normal. Laryngoscopy revealed hypertrophic vocal cords with normal movement. Patient II-6 had a milder presentation in early childhood with mildly itchy, erythematous maculopapular skin eruptions over both extremities that developed gradually into hyperpigmented, verrucous areas over elbows, knees, and knuckles. Both had smallhands and digits. They had mild microcephaly with a saddle nose and eyelid thickening (moniliform blepharosis). Muscle mass and strength were normal, but both had mild lower extremity spasticity with modest asymmetric hyperreflexia but without dystonic changes. Gait was grossly normal.

**Figure 2:**
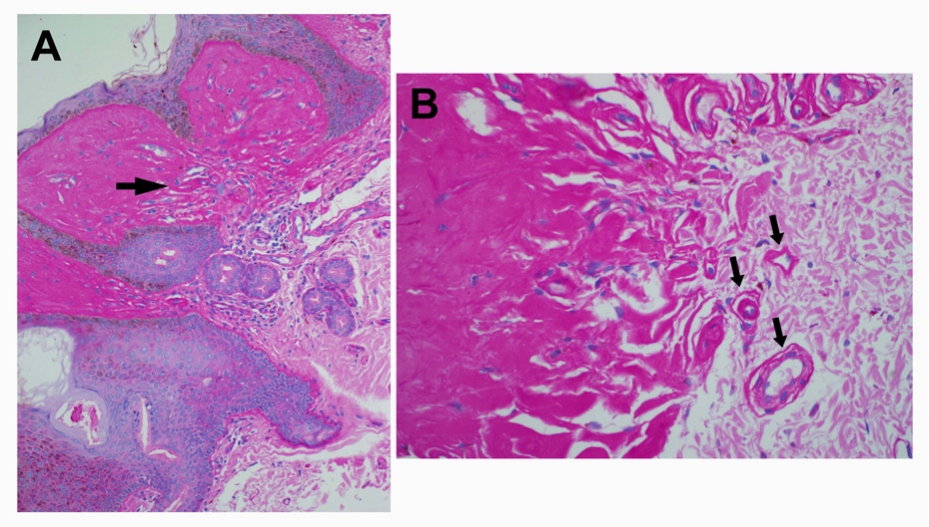
**Papillary dermis**. (a) Deposition of PAS positive and diastase-resistant material from Patient II-1 of Family 1. Rare inflammatory cells are seen in the upper dermis (original magnification X 200). (b) PAS positive material deposited around blood vessels which have thickened and hyalinized walls. (arrows, original magnification X 400). There was no evidence of infarction.

Skin biopsies revealed extensive deposition of amorphous eosinophilic, hyaline material characteristic of LP consisting of thick, homogenous bundles extending perpendicular to the skin surface. These bundles surrounded blood vessels in the thickened papillary dermis, and this material was PAS positive and diastase resistant (Figure 2). There were no ischemic changes around blood vessels.

**Figure 3:**
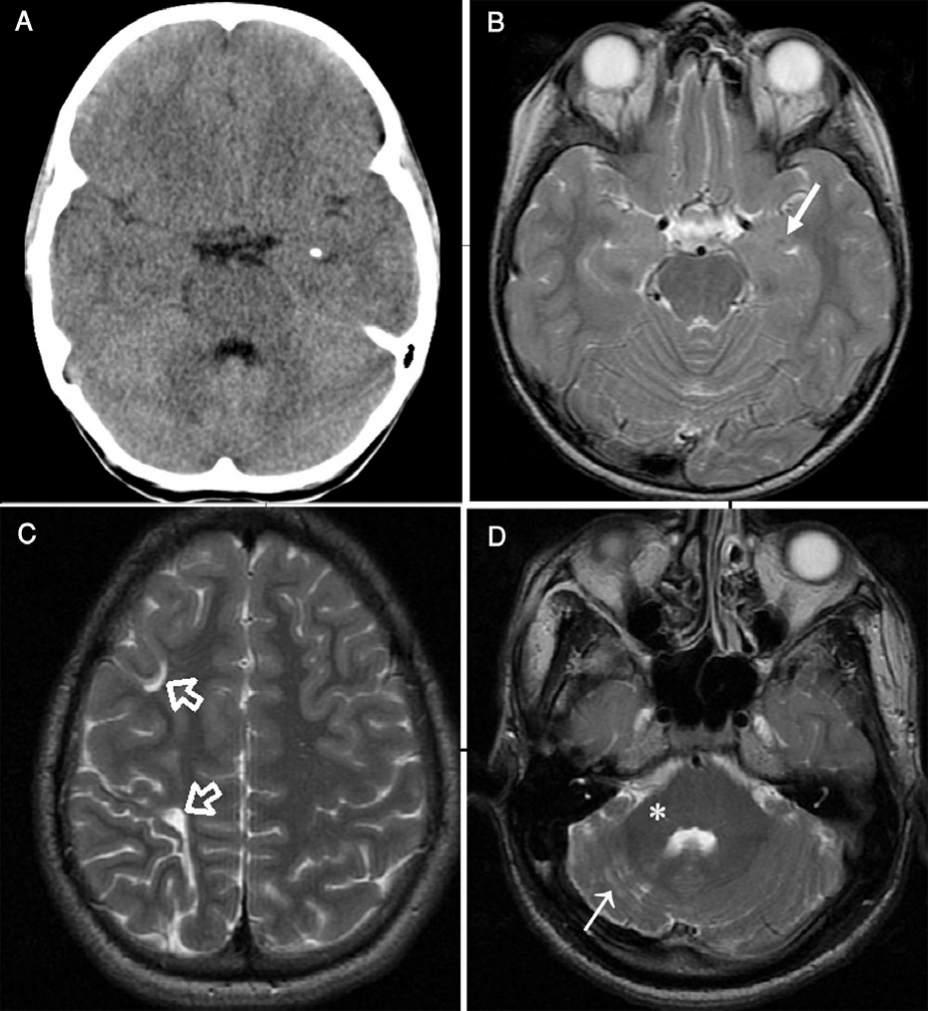
**Brain CT and MRI of Patients 1 and 2**. (a) Brain CT image of Patient II-6 of Family 1 at age of 10 years showing a small calcification in the left amygdale. Calcification in the right amygdale was seen on another section (not shown). (b) Brain axial T2-weighted MR image of the same patientat the level of temporal lobes showing a tiny hypointensity (arrow) representing amygdala calcification. The mesial temporal lobes were otherwise normal. (c) Brain axial T2-weighted MR images of Patient II-1 of Family 1 showing enlarged sulci and small gyri in the watershed zones of the right frontal lobe (open arrows) associated with loss of white matter due to old ischemia. (d) The right cerebellar hemisphere of the same patientwas small with widened folia, white matter gliosis (arrow), and a small right middle cerebellar peduncle (asterisk). Remodeling of the posterior fossa confirmed the long-standing nature of this abnormality. CT and MRI scans on patients from Families 2 and 3 were normal.

Brain computed tomography (CT) of Patient II-6 at age 10 years showed small calcifications in the amygdala bilaterally (Figure 3a). CT and magnetic resonance imaging (MRI) scans were otherwise normal with no evidence of atrophy or ischemia (Figure 3b). CT of Patient II-1revealed no brain calcification; however, both CT and MRI showed a watershed ischemic injury involving the right cerebral hemisphere with periventricular white matter loss (Figure 3c) and thickening of the overlying skull implying chronicity. He also had atrophy of the right cerebellar hemisphere and middle cerebellar peduncle (Figure 3d).

In general, patients from Family 2 (Figure 1b) were slightly more affected than patients from Family 3 (Figure 1c), although both families were less affected than Family 1. Rough, yellowish-white papular deposits in the skin and the oral mucosa were presentwithin the first year of life. They all developed multiple, small, severe blisters over the fingers of both hands leading to excoriations and post inflammatory hyperpigmentation mainly over joints with mild facial skin changes but no striking scarring. They all had beaded papules on the upper and lower labial mucosa and tongue together with multiple, small, waxy, yellow beaded papules along the eyelids (moniliform blepharosis). Hoarseness and pharyngeal involvement were present but also less evident.

Individuals from Families 2 and 3 had no developmental delay, and head circumference was normal in each. They were not mentally retarded, and each attended age-appropriate grades in normal schools, except for the youngest, who was not yet in school, and the girl from Family 3 (II-3), who dropped out of school, possibly for emotional reasons. Basic neurologic examinations were normal with no reflex or gait abnormality. CT and MRI scans (including Susceptibility-Weighted sequences, which are particularly sensitive to calcification and iron deposition) in each affected individual, except for Patient II-6 of Family 2, showed no temporal lobe calcification and no other brain abnormalities.

## Discussion

These seven patients alldeveloped characteristic skin lesions and hoarseness in infancy and had unequivocal LP by skin biopsy. Two individuals from Family 1 had modest mental retardation, and one had small amygdala calcifications, but the other affected patients had no definitive neurologic involvement when last examined between ages five and 19 years. All seven individuals had homozygous mutations in the *ECM1 *gene[3,12,13];Families 1 and 2 had novel *ECM1 *mutations, while Family 3 had a previously reported large deletion[2].

Innate severity of LP phenotypic expression is difficult to gauge for a number of reasons, even in carefully evaluated patients. Skin manifestations are recognized to be variable within families and even from time to time within the same individual, generally increasing with age and skin exposure, which is often determined by environment and employment. Pharyngeal involvement is usually obvious because of hoarseness and fibrosis involving the tongue and pharyngeal tissue, but these changes are difficult to quantify. Overt mental retardation may be straightforward to recognize, as in two of our patients, but more subtle intelligence changesmay not be easy to detect and may not be reported by families wishing to minimize their own impression of the impact of this genetic disease. Detecting subtle emotional or personality changes requires formal, quantitative neuropsychological testing standardized to the appropriate language and ethnic group. Such testing is not often available, as in the case of the families reported here. These families also demonstrate that current quality brain CT and MRI may be entirely normal in the temporal lobes and elsewhere even in teenage years and even with some indication of emotional difficulties (e.g., Patient II-3of Family 3).

LP has been reported to cause a variety of neurologic problems, including psychosis[9], partial complex seizures[6], emotional incontinence including rage and panic attacks[14], and progressive memory loss[7], most commonly of memories with emotional content[8]. Mental retardation has been reported in approximately one third of individuals with LP [6,9,15]and was documented in two of these seven individuals. The relative infrequency of other obviousneurologic abnormalities may be due tochance in a small series of patients or to the subtlety of changes in cognitive function or personality. Alternatively, patients with advanced or obvious neurologic abnormalities may have been over-represented in classic reports, or neuropsychological changes may be more frequent in certain well-studied populations (e.g., South African) than in others[16].

The etiology of mental retardation in LP remains uncertain because currently there is no information about *ECM1 *expression in human brain outside of blood vessels[12]. The *ECM1 *gene is an integral part of the extracellular matrix and basement membrane structure. These roles likely lead directly to the classic skin and pharyngeal damage when *ECM1 *is mutated [3], but the relevance to brain is less obvious. *ECM1 *interacts with other proteins making up the extracellular matrix [17,18], often regulating bioactivity of the binding partner[11], includingmatrix metalloproteinase 9 [19], which has an important role in temporal lobe synaptic physiology [20]. *ECM1 *mutations, therefore, might affect brain function, compromise homeostasis, or impair repair mechanisms independent of ischemia [19,21].

The brain pathophysiology of *ECM1 *has been hypothesized to result from a defect in cerebrovascular basement membrane that causes fibrinoid changes and calcification of vessel walls [8,15,22]. However, available neuropathology is limited, is from older individuals, and leaves open the question of whether the vascular pathology described is primary or secondary to some other process. Careful review of CT and MRI images yielded no hint of brain damage, including in the area immediately contiguous to temporal lobe calcification[14]in previously reported patients [4,8,14,23] or in Patient II-6 of Family 1. Therefore, a cerebral vasculopathic mechanism is not supported by the absence of ischemic changes on CT or MRI neuroimaging or in skin biopsies[6]. Even if ischemia were the cause of temporal lobe calcification, it is still unclear why this process should affect a particular area of the mesial temporal lobe and, when advanced, cause a fairly consistent comma-shaped area of calcification[24].

Patients from Family 1 were more affected in terms of skin, pharyngeal, and neurologic involvement than patients from Families 2 and 3, suggesting that severity ofintegumentinvolvement may roughly correspond to severity of brain involvement in certain LP individuals and families. Hamada and colleagues noted that individuals with mutations outside of exon 7 seemed more affected than individuals with mutations involving exon 7 of the *ECM1 *gene[3]. Indeed, Family 1, with a mutation in exon 8, was more affected than Family 2, with a mutation in exon 7. However, Family 1 had a two nucleotide deletion at the end of exon 8, leading almost immediately to a premature stop codon and effectively truncating all transcripts at that point. Family 3 had a large deletion removing exons 9 and 10 and accomplishing a comparable genetic defect. Nevertheless, skin, pharynx, and nervous systemof Family 1 seemedmore affected than in Family 3.

Most genetic reports regarding LP have observed that phenotype-genotype correlations are not obvious[3,25], and this seems also to be true in our three families. The *ECM1 *gene has several transcripts, each of which may be differentially active in various tissues, including various parts of the brain, at different times during an individual's lifetime, offering the possibility of substantial variability in the effect of mutations in particular individuals. In addition, novel transcripts may provide partial rescue through in-frame skipping of mutated exons in certain circumstances[3]. This genetic complexity is currently difficult to either predict or assess in individual patients. Similarly, our current ability to completely quantify clinical, including neurologic, involvement is limited. Taken together, these factors mean that the lack of phenotype-genotype correlation is not surprising. Therefore, a suspected diagnosis of LP should be confirmed by sequencing *ECM1 *exons 6 and 7 first because this is where most mutations occur[3], but homozygous or compound heterozygous mutations of any type may occur in any exon.

## Conclusions

These individuals illustrate the neurologic spectrum of LP, including variable mental retardation, personality changes, and mesial temporal calcification but imply that significant neurologic involvement may be somewhat less common that previously thought. The cause of neurologic abnormalities was not clear from either neuroimaging or from what is known about *ECM1 *function. The severity of dermatologic abnormalities and hoarseness generally correlated with neurologic abnormalities, with Family 1 being somewhat more affected in all spheres than the other two families. Nevertheless, phenotype-genotype correlation was not obvious, possibly because of difficulty quantifying the neurologic phenotype and because of genetic complexity.

## Abbreviations

CT: Computed tomography; ECM1: Extracellular matrix protein 1; LP: Lipoid proteinosis; MRI: magnetic resonance imaging; UTR: Untranslated Region

## Competing interests

The authors declare that they have no competing interests.

## Authors' contributions

MASevaluated clinical and neurologic features of the patients; IAO was in charge of the radiological evaluation; TMB evaluatedneurologic and neuro-ophthalmologic features; DTO evaluated ocular motility;LL, JAM, and LVM evaluated additional dermatologic and clinical features; and YHF and AHS evaluated ophthalmological features. KKA was in charge of analysis of genetic data and writing the genetic portion of the manuscript. KKA and TMB were responsible for study design and manuscript completion. All authors read and approved the final manuscript.

## Pre-publication history

The pre-publication history for this paper can be accessed here:

http://www.biomedcentral.com/1471-2350/12/31/prepub
